# WDHD1 is over‐expressed in nasopharyngeal carcinoma and may control the expression of ITGAV

**DOI:** 10.1002/2211-5463.13519

**Published:** 2022-11-28

**Authors:** Ji‐Yun Wu, Yi‐Tong Niu, Su‐Ning Huang, Yu‐Min Tan, Zhen‐Dong Yang, Ye‐Ying Fang, Li Jiang, Ting‐Ting Zhang, Xiao‐Fen Zeng, Yun‐Xi Peng, Miao Mo, Cai‐Xing Lin, Zhu‐Xin Wei

**Affiliations:** ^1^ Department of Radiotherapy First Affiliated Hospital of Guangxi Medical University Nanning China; ^2^ Department of Radiotherapy Guangxi Medical University Cancer Hospital Nanning China; ^3^ Department of Otolaryngology First People's Hospital of Hechi City Yizhou China

**Keywords:** bioinformatics, cell cycle, immunohistochemistry, nasopharyngeal carcinoma, WD repeat and HMG‐box DNA binding protein 1

## Abstract

Nasopharyngeal carcinoma (NPC) is a highly metastatic and invasive malignant tumor that originates in the nasopharynx. The DNA‐binding protein WD repeat and HMG‐box DNA‐binding protein 1 (WDHD1) are highly expressed in a variety of tumours, but its expression and mechanism of action in NPC have not been reported to date. To investigate the involvement of WDHD1 in NPC, we first mined databases for the gene expression profile of NPC. Immunohistochemistry (IHC) was performed on 338 cases of NPC and 112 non‐NPC samples to verify the results. We report that the expression of WDHD1 is significantly elevated in NPC. ChIP‐seq was used to show that integrin alpha V (ITGAV) and WDHD1 exhibit a significant binding peak in the promoter region of the ITGAV gene. The expression levels of ITGAV and WDHD1 exhibit a significant positive correlation, and IHC was performed to show that ITGAV is highly expressed in NPC. Expression of ITGAV increased after overexpression of WDHD1, suggesting that ITGAV may be a potential target gene of WDHD1. Pathway analysis showed that both genes were closely related to the cell cycle, and flow cytometry was used to further confirm that decreased expression of WDHD1 significantly increased the number of apoptotic cells. In conclusion, our results suggest that expression of WDHD1 is increased in NPC and is likely to be associated with the NPC cell cycle; thus, we propose that WDHD1 may have the potential as a target gene for primary screening and treatment of NPC.

## Graphical abstract

Comprehensive analysis showed that WDHD1 is highly expressed in nasopharyngeal carcinoma (NPC) tissues, and it may affect cell apoptosis by regulating the expression of ITGAV. Our data suggest that WDHD1 may play a role in the malignant progression of NPC.

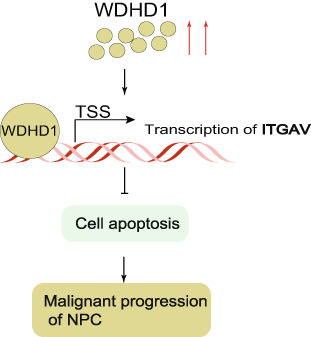

**Abbreviations**
AUC: the area under the curve
Cistrome DB: Cistrome Data Browser
DEG: differentially expressed gene
EMT: epithelial‐mesenchymal transformation
GEO: Gene Expression Omnibus
GO: Gene Ontology
HCC: hepatocellular carcinoma
IGV: Integrative Genomics Viewer
ITGAV: integrin alpha V
KEGG: Kyoto Encyclopedia of Genes and Genomes
NPC: nasopharyngeal carcinoma
NSCLC: non‐small cell lung cancer cells
SAC: spindle assembly checkpoint
sROC: summary receiver operating characteristic curve
TCGA: The Cancer Genome Atlas
WDHD1: WD repeat and HMG‐box DNA‐binding protein 1

Nasopharyngeal carcinoma (NPC) is a highly metastatic and invasive malignant tumour that originates in the nasopharynx. Available statistics show that 133 354 new cases and 80 008 deaths from NPC occurred worldwide in 2020 [1], indicating the serious danger of this cancer to human health. The incidence of NPC has clear regional characteristics, with 70% of new cases occurring in Southeast and East Asia, especially in Guangdong and Guangxi in China [2]. In the recent years, the age of onset has become younger and younger, imposing a heavy economic burden on patients' families and society. Radiotherapy is still the preferred treatment for NPC due to its hidden location and sensitivity to radiation. However, in areas with a high incidence of NPC, about 70% of patients are already in an advanced stage when diagnosed [3], and clinical intervention is late. For these reasons, the overall prognosis is poor and the recurrence rate is high. Clarifying the specific pathogenesis of NPC and finding new diagnostic and therapeutic targets are goals that require further efforts.

WD repeat and HMG‐box DNA‐binding protein 1 (WDHD1) is a DNA‐binding protein with a total molecular weight of 125 kDa, composed of 1127 amino acids [4]. Extensive research has shown that WDHD1 is associated with the cell cycle. Its possible mechanisms of action may involve: (a) regulation of entry into the S phase, as WDHD1 depletion leads to G1 stagnation and delays progression to the S phase [5, 6]; (b) regulation of the assembly and activation of the pre‐replication complex (pre‐RC), two key steps in the initiation of DNA replication [7]; and (c) control of the repair process to correct base mismatch and insertion‐deletion mismatch during DNA replication [8]. For these reasons, WDHD1 is implicated in the development of many cancers. TCGA analysis and genome‐wide siRNA screening performed by Ertay et al. [9] showed a higher expression of WDHD1 mRNA in triple‐negative breast cancer than in normal breast tissue, with expression related to tumour size, proliferation, and poor prognosis [9]. Other studies have shown overexpression of WDHD1 in the esophageal, lung, bile duct, and cervical cancers [10, 11, 12], as well as an association with poor prognosis and malignant progression. However, WDHD1 expression and its effects have not yet been reported for NPC.

In our research, we searched The Cancer Genome Atlas (TCGA) (https://cancergenome.nih.gov/), Gene Expression Omnibus (GEO) (http://www.ncbi.nlm.nih.gov/geo/), ArrayExpress database (http://www.ebi.ac.uk/arrayexpress/), and related literature, followed by combined bioinformatic and immunohistochemical analysis to obtain a comprehensive picture of WDHD1 expression in NPC. We also predicted the downstream target gene of WDHD1, and explored the expression of the predicted target gene in NPC by immunohistochemistry (IHC). PCR was used to analyze the relationship between their expressions. In addition, possible molecular functions and signaling pathways were forecasted by pathway analysis, and the results were preliminarily verified by flow cytometry apoptosis assay (Fig. 1A). Our study provides a basis for developing new diagnostic markers and therapeutic targets for NPC.

**Fig. 1:**
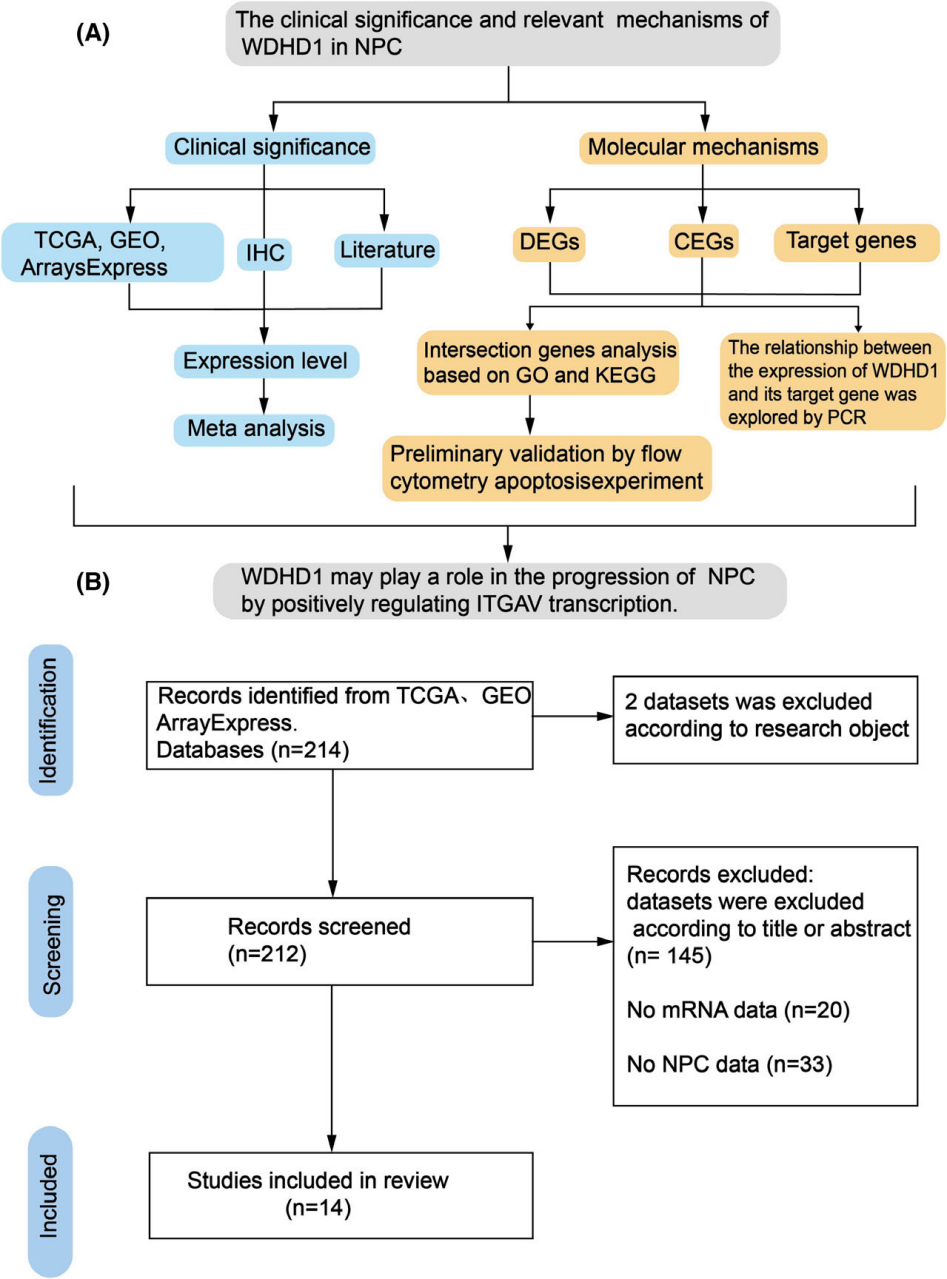
Flow charts of the study design and datasets selection process. (A) Flow chart of the study design; (B) flow chart of datasets selection process. CEGs, co‐expressed genes.

## Materials and methods

### Bioinformatics data mining and processing

Nasopharyngeal carcinoma‐related datasets were downloaded from the GEO databases (Fig. 1B). We searched for “(nasopharyngeal OR nasopharynx) AND (neoplasm OR cancer OR carcinoma OR malignancy)” and included datasets according to the following criteria: (a) human NPC tissues were studied, (b) each dataset contained WDHD1 mRNA expression profile data, and (c) the experimental group contained no < 3 samples. The expressed data were processed in the R 4.0.2 software environment (Lucent Technologies, Jasmine Hill, NJ, USA), and fragments per kilobase of exon model per million mapped fragments (FPKM) from RNA‐seq experiments were converted to tethered particle motion (TPM) to eliminate the effects of gene length and sequencing depth on the expression level. The unstandardized data were converted by the formula log_2_(*x* + 0.001), followed by the use of the normalize Between Arrays function of the limma package to eliminate the inter‐sample batch effect in the same dataset. We also combined the same platform datasets and normalized the batch effect using the combat function of the sva package.

### Statistical analysis

After obtaining the expression data for WDHD1, we conducted an analysis of the expression data using the independent‐samples *t*‐test of ibm spss statistics version 25.0 (IBM Corp., Armonk, NY, USA) to confirm the significance of WDHD1 expression differences between NPCs and adjacent non‐NPCs (mean was used to describe the average level of this group of data; SD was used to describe the degree of dispersion). The data were also analyzed using stata 15.0 (Stata Corp LP, College Station, TX, USA) to increase the accuracy of the results and to evaluate the ability of WDHD1 expression to distinguish between NPCs and non‐NPCs. The *I*‐square (*I* ^2^) and chi‐square tests were used to determine the level of heterogeneity between different datasets when drawing forest maps. A value of *P* < 0.05 or *I* ^2^ > 50% indicated significant heterogeneity, and a random effect model was chosen for analysis. We used both Begg's and Egger's methods for funnel plots to evaluate publication bias, with a value of *P* > 0.05 suggesting that publication bias was not significant. A summary receiver operating characteristic curve (sROC) was drawn, and the area under the curve (AUC) was used to measure the ability of WDHD1 to differentiate non‐cancerous tissue from NPC.

### Immunohistochemistry

Tissue microarrays were prepared by Pantomics, Inc. (Richmond, CA, USA) from 338 NPC tissues and 112 nasopharyngeal mucosa chronic inflammation tissues (NPC TMA1, NPC TMA2, NPC131, NPC241 and NPC482). Paraffin‐embedded sections were prepared and deparaffinized using conventional procedures. After hydration, tissue sections were placed in ethylene diamine tetraacetic acid (EDTA) antigen retrieval solution for antigen repair, followed by 3% H_2_O_2_ blocking endogenous peroxidase. The primary antibody (rabbit polyclonal antibody of WDHD1, ab224221, Abcam (Cambridge, MA, USA); rabbit monoclonal antibody of ITGAV, ab179475, Abcam) was diluted (Zhong Shan Golden Bridge Biotechnology, Beijing, China) at a ratio of 1 : 100 and then incubated at 37 °C for 70 min. The rest of the steps were performed according to a previously reported procedure [13]. Staining was assessed under a light microscope, and we used two different methods to assess the IHC results of WDHD1 and ITGAV. For WDHD1, we randomly selected five non‐overlapping high‐magnification fields, and calculated the number of positive cells per 100 cells in each field. The final result was the mean number of the five fields. For ITGAV, the final total score was the product of the percentage of positive cells and the staining intensity. The percentage of positive cells was evaluated as follows: 0 (< 5%); 1 (6–25%); 2 (26–50%); 3 (51–75%); and 4 (> 76%). The staining intensity score criteria were as follows: 0 (colourless); 1 (light yellow, mild staining); 2 (tan, medium staining); and 3 (brown, strong staining). We obtained a more intuitive display of the differential expression of WDHD1 and ITGAV in control tissues and experimental tissues by drawing a violin diagram. We then used stata 15.0 to draw the ROC curve to evaluate the ability of WDHD1 and ITGAV to distinguish between NPC and non‐NPC tissues. In addition, the clinicopathological parameters of the patients were collated, and the statistical differences among the groups were estimated using the Student's *t*‐test.

### Potential transcriptional mechanisms of WDHD1 in NPC

To obtain a better understanding of the mechanism of WDHD1 in NPC, we gathered the following three gene sets (WDHD1 target genes, co‐expressed genes of WDHD1 and NPC differentially expressed genes) to predict the potential target gene of WDHD1 with their intersection. We first downloaded all target genes of WDHD1 from the Cistrome Data Browser (Cistrome DB), and selected putative target genes with scores ≥ 1 for further analysis. We then used r 4.0.2 and the Pearson correlation algorithm to screen the co‐expressed genes of WDHD1 (CEGs), and collected genes with correlation coefficients ≥ 0.3, *P*‐values < 0.05, and presence in more than two datasets. We then screened differentially expressed genes (DEGs) with the limma package, and we collected genes with log_2_FoldChange > 1, *P* < 0.05 and presence in more than two datasets. Some up‐regulated and positively correlated putative target genes were obtained using the intersection of the three gene screens. We then selected a gene that had significant binding peaks with WDHD1, downloaded the bigwig files, and imported them into the integrative genomics viewer (igv) (Cambridge, MA, USA) to draw a peak graph. We further explored the relationship between the predicted target gene and WDHD1 and its expression in NPC tissues by plotting the sROC curve and correlation scatter plots based on GEO databases and IHC, and calculating the SMD value.

PCR was used to detect the expression of ITGAV after overexpressed the expression of WDHD1 in C666‐1 and IHC was used to further verify the expression of the predicted target in NPC tissues (NPC TMA1, including 96 NPC cases and 30 non‐NPC cases). We used the clusterprofiler r Package to obtain a deeper understanding of the biological function and mechanism of WDHD1 in NPC by conducting Gene Ontology (GO) and Kyoto Encyclopedia of Genes and Genomes (KEGG) enrichment analyses on the intersection of the genes collected above and ITGAV‐related DEGs (the intersection of ITGAV positive‐related genes and NPC DEGs; The screening method of ITGAV positively related genes was consistent with that of WDHD1 positively related genes, and the Pearson correlation algorithm was used to screen them in r 4.0.2. The screening criteria were correlation coefficients ≥ 0.3, *P*‐values < 0.05, and presence in more than four datasets; DEGs appeared in no less than three datasets. We then used the intersection of the two gene screens for further analysis).

### Lentivirus infection

The concentrated virus solution was purchased from Shanghai Jikai Gene Chemical Technology Co., Ltd (Shanghai, China), and stored at −80 °C (the component sequence of the WDHD1 overexpression group was Ubi‐MCS‐3FLAG‐CBh‐gcGFP‐IRES‐puromycin, GV492; the negative control was constructed with empty vectors, and nonsense sequences were not inserted, CON335). C666‐1 cells with good‐growing conditions in the logarithmic growth phase were taken and inoculated in 6‐well plates with 2 × 10^5^ cells per well. The cells were infected after adherence on the second day. The corresponding amount of virus was added according to MOI = 75, and 40 μL of transfection reagent HitransG A was added. The complete medium was supplemented to 1 mL per well, and the culture was continued for 15 h in a 37 °C, 5% CO_2_ incubator. The transfection was observed under an inverted fluorescence microscope 72 h after infection. If the cell growth condition is good and the degree of fusion is more than 70%, the medium containing puromycin can be used to screen the infected cells (the initial screening concentration is 3 μg·mL^−1^, which is changed to 1 μg·mL^−1^ for maintenance culture for about a week). The fluorescence was observed under an inverted fluorescence microscope, and when the fluorescence efficiency was almost 100%, the cells were expanded and collected for qPCR.

### siRNA transfection

CNE‐2 cells in the logarithmic growth phase and a good growth state were inoculated in 6‐well plates at 2 × 10^5^ cells per well and transfected after cell adhesion (the target sequence of WDHD1 siRNA: 5′‐GCUGUGAAUUUAGCCAUUATT‐3′; and the sequence of negative control was confidential, which did not target any gene product. Both were purchased from Huzhou Hippo Biotechnology Co., Ltd [Huzhou, Zhejiang, China]). At 2 h before transfection, the culture medium was washed and then replaced with serum‐free 1640 medium, and transfection was carried out according to the instructions. After 6 h, the medium was exchanged with 2 mL of fresh complete medium, and the cells were incubated for 48 h in a constant‐temperature incubator (37 °C, 5% CO_2_).

### RNA extraction, reverse transcription and real‐time fluorescence quantitative PCR

We extracted the total RNA with TRIzol reagent (Shanghai Pufei Biotechnology Co., LTD, Shanghai, China) according to the instructions, and then reverse‐transcribed the RNA into cDNA using a Promega M‐MLV kit (Promega (Beijing) Biotech Co., Ltd, Beijing, China). The product was evaluated with light absorption at 260 and 280 nm using a spectrophotometer. Primer sequences were designed, and all the primers were synthesized by Sangon Biotech (Shanghai) Co., Ltd (Shanghai, China). The primer sequences were as follows: GAPDH (F): 5′‐TGACTTCAACAGCGACACCCA‐3′, (R): 5′‐CACCCTGTTGCTGTAGCCAAA‐3′; WDHD1 (F): 5′‐GGGAAGCAGCTCTTGTGGAT‐3′, (R): 5′‐GTGTGTCCCTCTGTATGCCC‐3′; ITGAV (F): 5′‐TCTGTGGCTGTCGGAGATTTCAATG‐3′, (R): 5′‐TTCCCAAAGTCCTTGCTGCTCTTG‐3′. The PCR reaction system was then used for amplification on an ABI 7500HT system (Thermo Fisher Scientific, Waltham, MA, USA). SYBR‐Green analysis was used for quantitative detection of gene expression, and $2^{-\Delta \Delta C_{T}}$ was used for calculation (Δ*C*_T_ = *C*_T_ value of target gene − *C*_T_ value of reference gene GAPDH, −∆∆*C*_T_ = ∆*C*_T_ of the control group − ∆*C*_T_ of experimental group). Finally, we used an independent‐samples *t*‐test to determine whether the difference between the negative control group and the experimental group was statistically significant.

### Cell apoptosis detection by flow cytometry

After 48 h of culture, 0.25% trypsin without EDTA was added to digest the transfected CNE‐2 cells. The cells were washed twice with phosphate buffered saline, centrifuged at 1500 ***g*** for 3 min, and the supernatant was removed. An Annexin V‐FITC/PI apoptosis detection kit (Tianjin Sungene Biotech Co., Ltd, Tianjin, China) was used as follows: 500 μL binding buffer was added to resuspend the cells, followed by 5 μL Annexin V‐FITC and 5 μL PI. The tube was mixed well and then incubated and protected from light at room temperature for 5–15 min. Cell apoptosis was detected using flow cytometry and statistically analyzed by an independent‐samples *t*‐test (the upper right quadrant should be the late apoptotic cell, and the lower right quadrant should be the early apoptotic cell). All the experiments were repeated three times.

## Results

### The association between WDHD1 expression and NPC was analyzed based on online database information

**Fig. 2:**
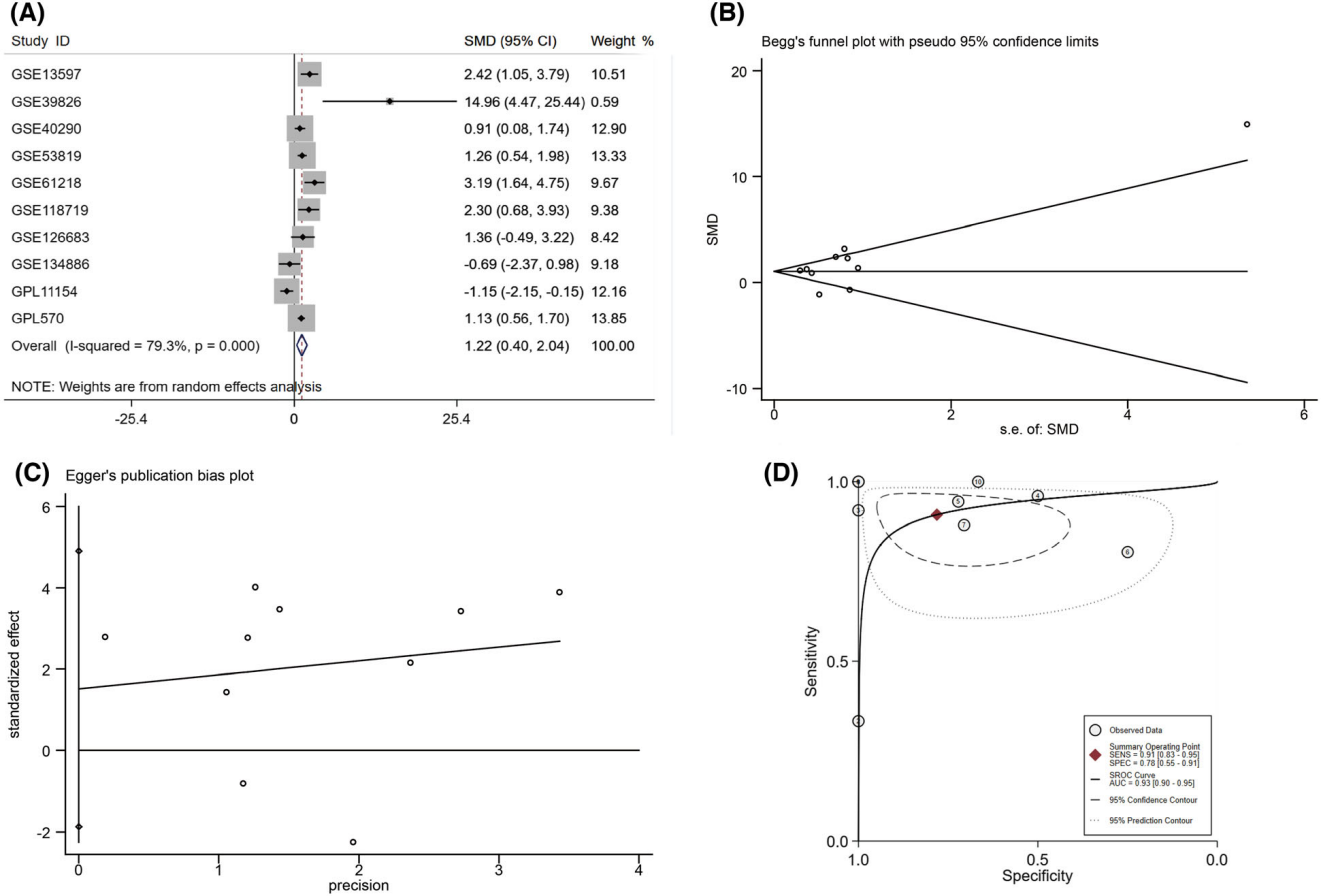
Overall WDHD1 expression of NPC groups and non‐cancerous groups in 14 datasets. (A) Forest profile of SMD of the expression of WDHD1 between NPCs and non‐NPCs; (B) Begg's funnel plot for publication bias test; (C) the Egger's publication bias plot; (D) sROC curve. SMD, standard mean deviation.

After screening, a total of 14 datasets (nine arrays: GSE13597, GSE40290, GSE53819, GSE61218, GSE126683, GSE39826, GSE64634, GSE34573 and GSE12452; and five RNA‐seq datasets: GSE118719, GSE68799, GSE63381, GSE102349 and GSE134886) were collected; datasets from the same platform were combined, and batch effects were removed using the combat function of the sva package (GSE68799, GSE63381 and GSE102349 were combined into GPL11154, while GSE64634, GSE34573 and GSE12452 were combined into GPL570). The WDHD1 expression data in each dataset were then extracted for comprehensive analysis. The values of mean ± SD revealed a significant expression of WDHD1 in NPC in most datasets (*P* < 0.05) (Table 1). The forest map showed high heterogeneity (*I* ^2^ = 79.3%, *P* < 0.001), so a random effect model was selected for analysis. The expression of WDHD1 mRNA was significantly higher in the NPC group than in the control group (SMD = 1.22, 95% CI = 0.40–2.04) (Fig. 2A). The Begg's test did not detect significant publication bias (*P* = 0.592, > 0.05) (Fig. 2B), nor did the Egger's test (*P* = 0.332, > 0.05) (Fig. 2C). The sROC curve showed an AUC = 0.93 [0.90–0.95], and the sensitivity and specificity were 0.91 [0.83–0.95] and 0.78 [0.55–0.91], respectively (Fig. 2D), indicating that WDHD1 had a strong ability to distinguish between NPC and non‐NPC tissues.

**Table 1:** Overview of the 14 datasets selected from GEO database. SD, standard deviation; Mean1 ± SD1, NPC tissues; Mean0 ± SD0, non‐cancer tissues; GPL11154, includes GSE68799, GSE63381 and GSE102349; GPL570, includes GSE64634, GSE34573 and GSE12452; *T*, *T* value of Student's *t* test; *P*, *P*‐value of Student's *t*‐test.

Study	Country	Platforms	Cancer group	Control group	Mean1 ± SD1	Mean0 ± SD0	*T*	*P*
GSE13597	UK	GPL96	25	3	4.07 ± 0.39	3.13 ± 0.38	3.96	< 0.001
GSE39826	UK	GPL6244	3	3	8.54 ± 0.05	6.74 ± 0.16	18.32	< 0.001
GSE40290	China	GPL8380	25	8	−2.19 ± 0.56	−2.72 ± 0.65	2.24	0.032
GSE53819	China	GPL6480	18	18	7.83 ± 0.53	7.10 ± 0.63	3.77	< 0.001
GSE61218	China	GPL19061	10	6	6.23 ± 0.40	5.16 ± 0.17	6.18	< 0.001
GSE118719	USA	GPL20301	7	4	5.06 ± 0.60	3.84 ± 0.33	3.59	0.006
GSE126683	China	GPL16956	3	3	7.36 ± 0.17	7.03 ± 0.29	1.67	0.170
GSE134886	China	GPL20795	3	3	2.65 ± 0.57	2.94 ± 0.15	0.85	0.443
GPL11154	–	GPL11154	159	4	2.15 ± 0.43	2.65 ± 0.60	2.27	0.025
GPL570	–	GPL570	58	17	5.52 ± 0.10	4.38 ± 1.03	4.11	< 0.001
Total			311	69				

### IHC analysis of the expression of the WDHD1 protein in NPC

**Fig. 3:**
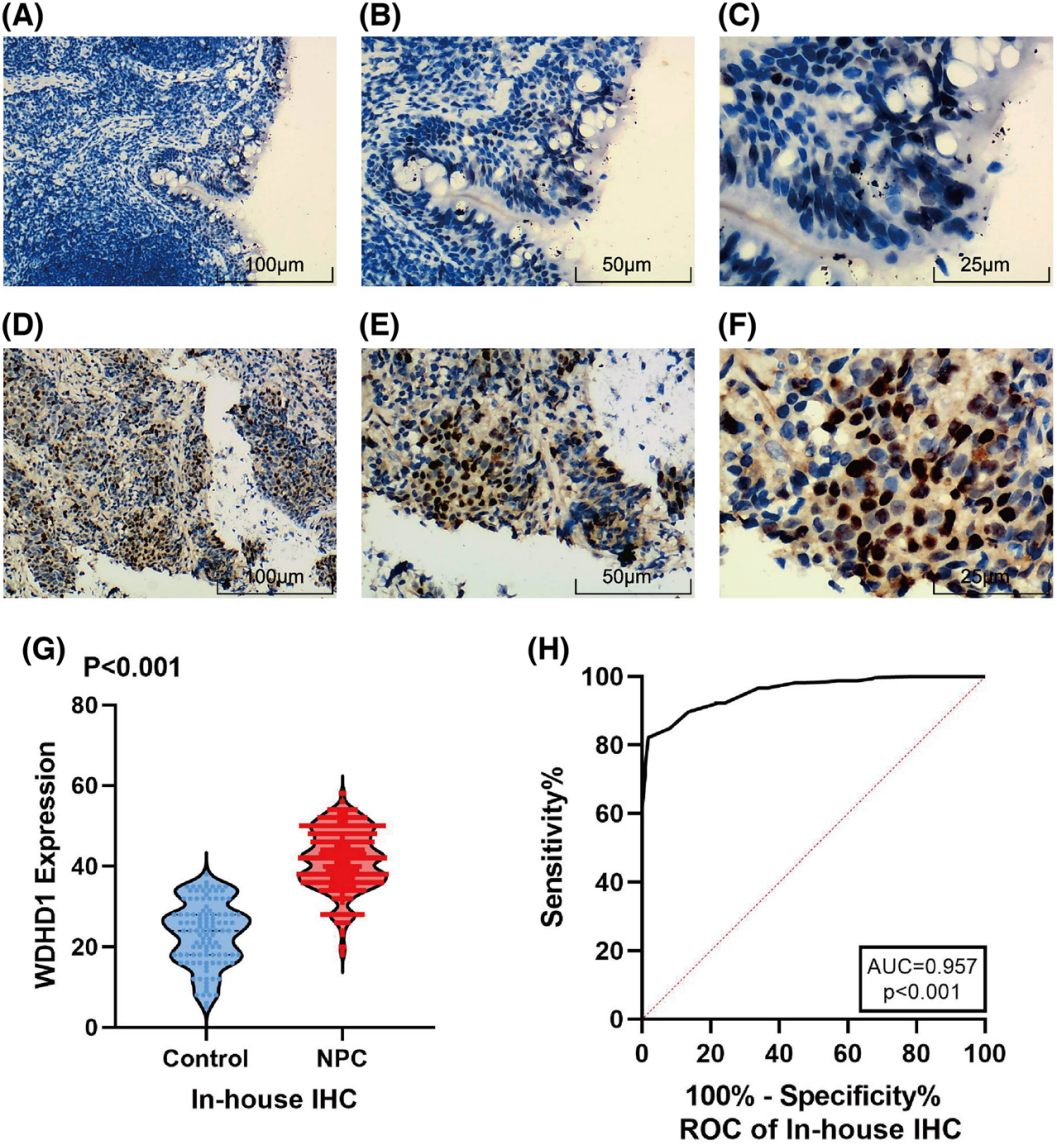
Expression of WDHD1 protein in NPC. (A–C) WDHD1 expression in non‐cancer tissues; (D–F) WDHD1 expression in NPC tissues. Magnification: ×100 (A, D) (scale bar = 100 μm), ×200 (B, E) (scale bar = 50 μm) or ×400 (C, F) (scale bar = 25 μm); (G) the expression of WDHD1 between non‐cancer and NPC based on IHC; (H) the ROC curve of WDHD1 based on IHC. ROC, receiver operating characteristic curve.

We also used IHC to explore the expression of the WDHD1 protein in NPC. Pathology sections of 338 NPC tissues and 112 nasopharyngeal mucosa with chronic inflammation were prepared. Expression of WDHD1 protein in NPC tissues showed nuclear localization (Fig. 3A–F) and was higher in the NPC tissues than in nasopharyngeal mucosa with chronic inflammation (*P* < 0.001) (Fig. 3G), and the ROC curve showed AUC = 0.957 (*P* < 0.001), indicating that WDHD1 has a strong ability to distinguish NPC from non‐NPC tissues (Fig. 3H), in agreement with the results of the bioinformatic analysis. A further statistical analysis of the clinicopathological information of the patients revealed that WDHD1 expression was not significantly correlated with age or gender (Table 2). Further investigation is needed regarding other pathological features.

**Table 2:** WDHD1 expression and clinicopathological features in NPC tissues based on IHC. SD, standard deviation; *T*, *T* value of Student's *t*‐test; *P*, *P*‐value of Student's *t*‐test.

Clinicopathological parameters	*N*	*M* ± SD	*T*	*P*
Tissues				
NPC	338	41.49 ± 7.49	21.95	< 0.001
Controls	112	23.30 ± 7.93		
Gender				
Male	257	41.70 ± 7.38	0.90	0.368
Female	81	40.84 ± 7.85		
Age (year)				
< 60	270	41.52 ± 7.48	0.137	0.891
≥ 60	68	41.38 ± 7.57		

### Analysis of the potential mechanism of WDHD1 in NPC

**Fig. 4:**
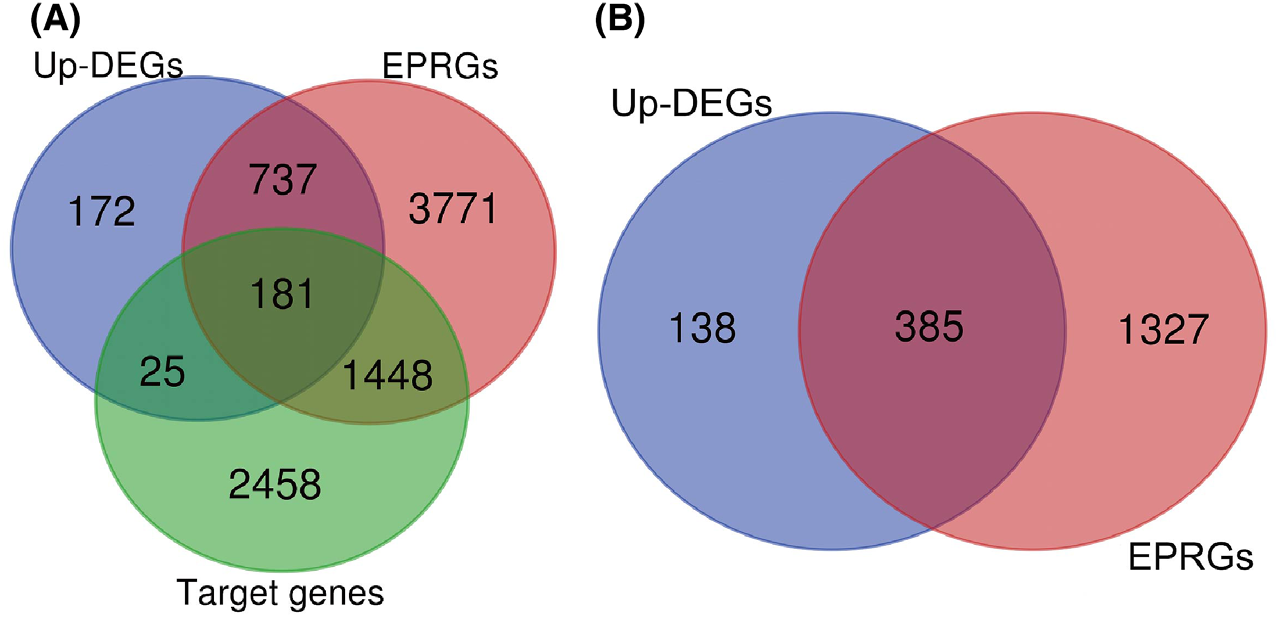
Venn diagram. (A) Venn diagram of WDHD1‐related DEGs (the intersection of EPRGs, up‐DEGs and target genes); (B) Venn diagram of ITGAV related DEGs (the intersection of EPRGs and up‐DEGs). EPRGs, express positively related genes.

**Fig. 5:**
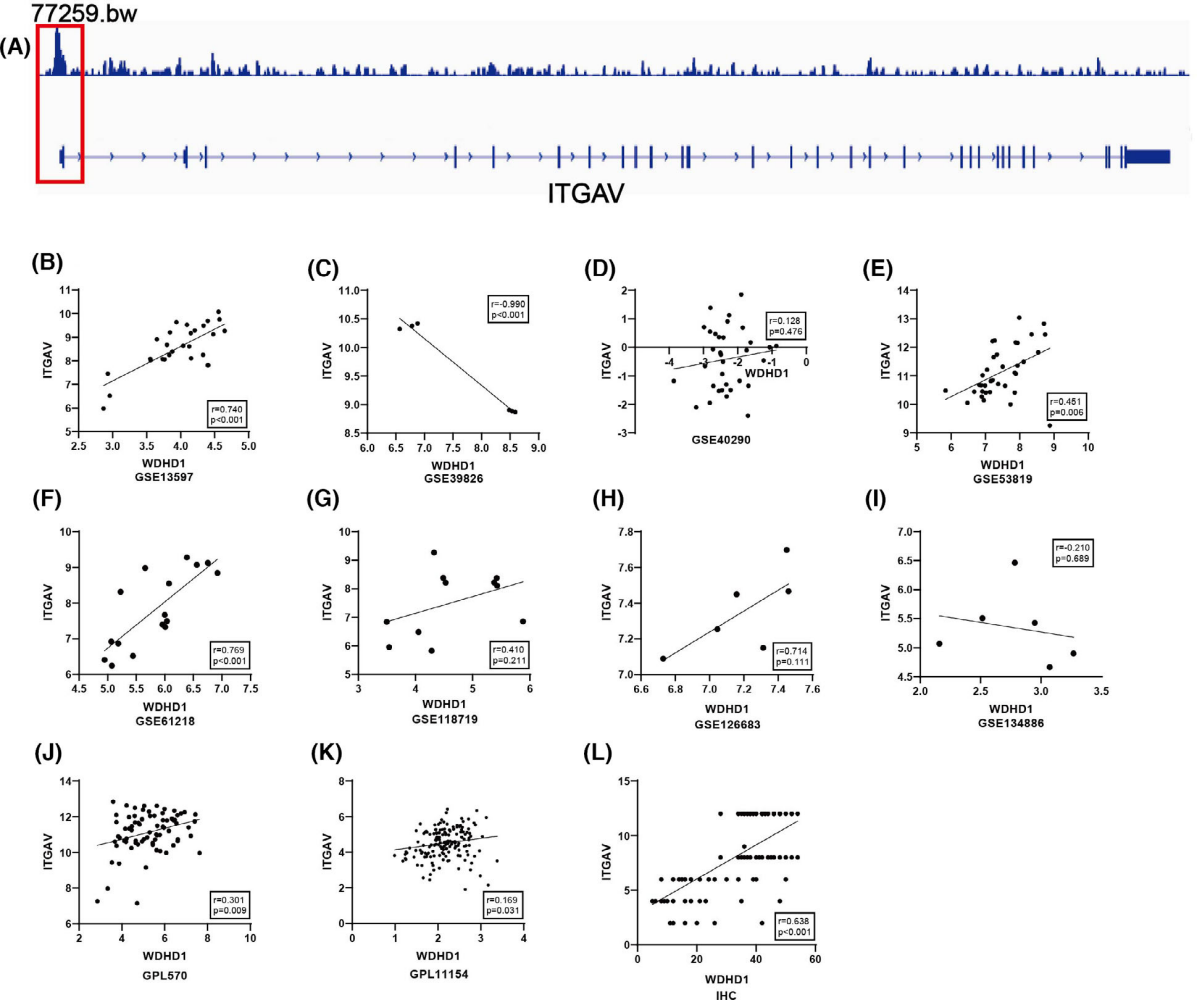
Relationship between transcription factor WDHD1 and its potential target gene ITGAV. (A) Chip‐seq show that WDHD1 binds to the promoter region of ITGAV; (B–L) scatter plots of correlation between WDHD1 and ITGAV based on GEO and IHC.

**Fig. 6:**
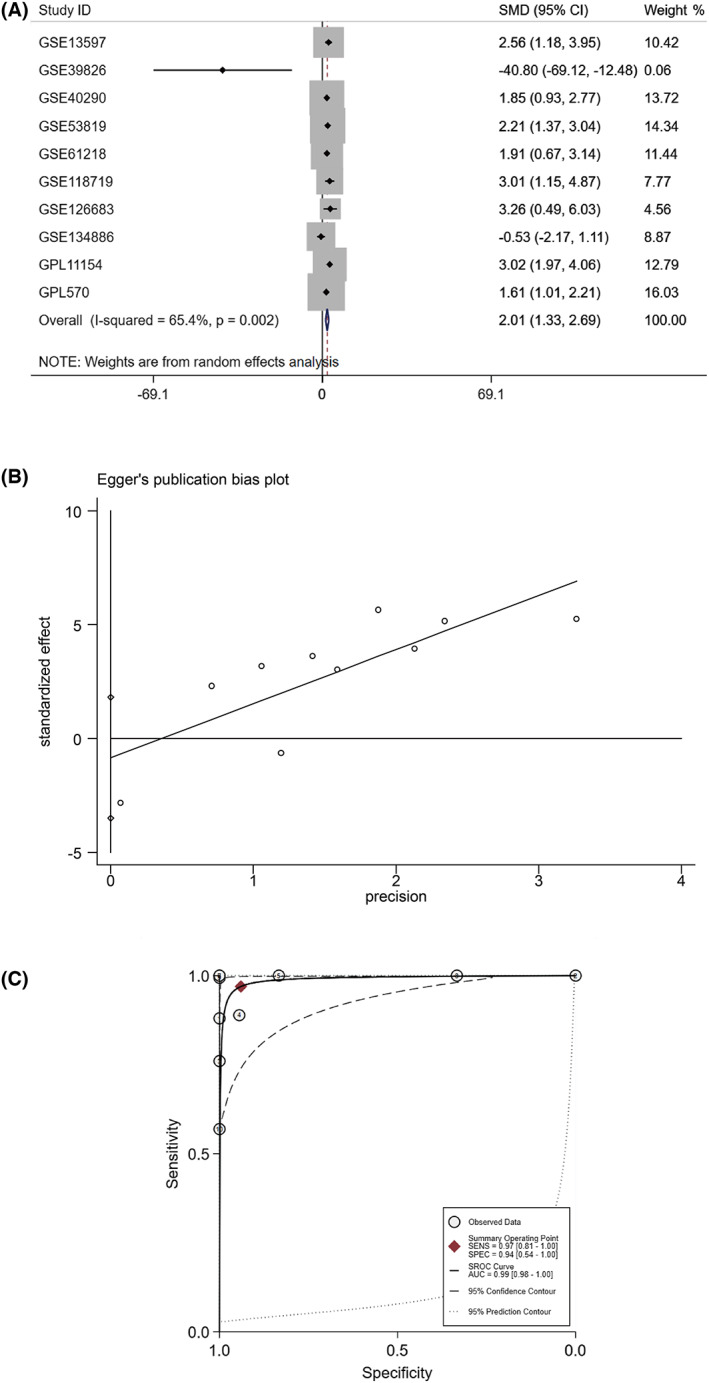
Overall ITGAV expression of NPC groups and non‐cancerous groups in 14 datasets. (A) Forest profile of SMD of the expression of ITGAV between NPCs and non‐NPCs; (B) Egger's funnel plot for publication bias test; (C) sROC curve. SMD, standard mean deviation.

**Fig. 7:**
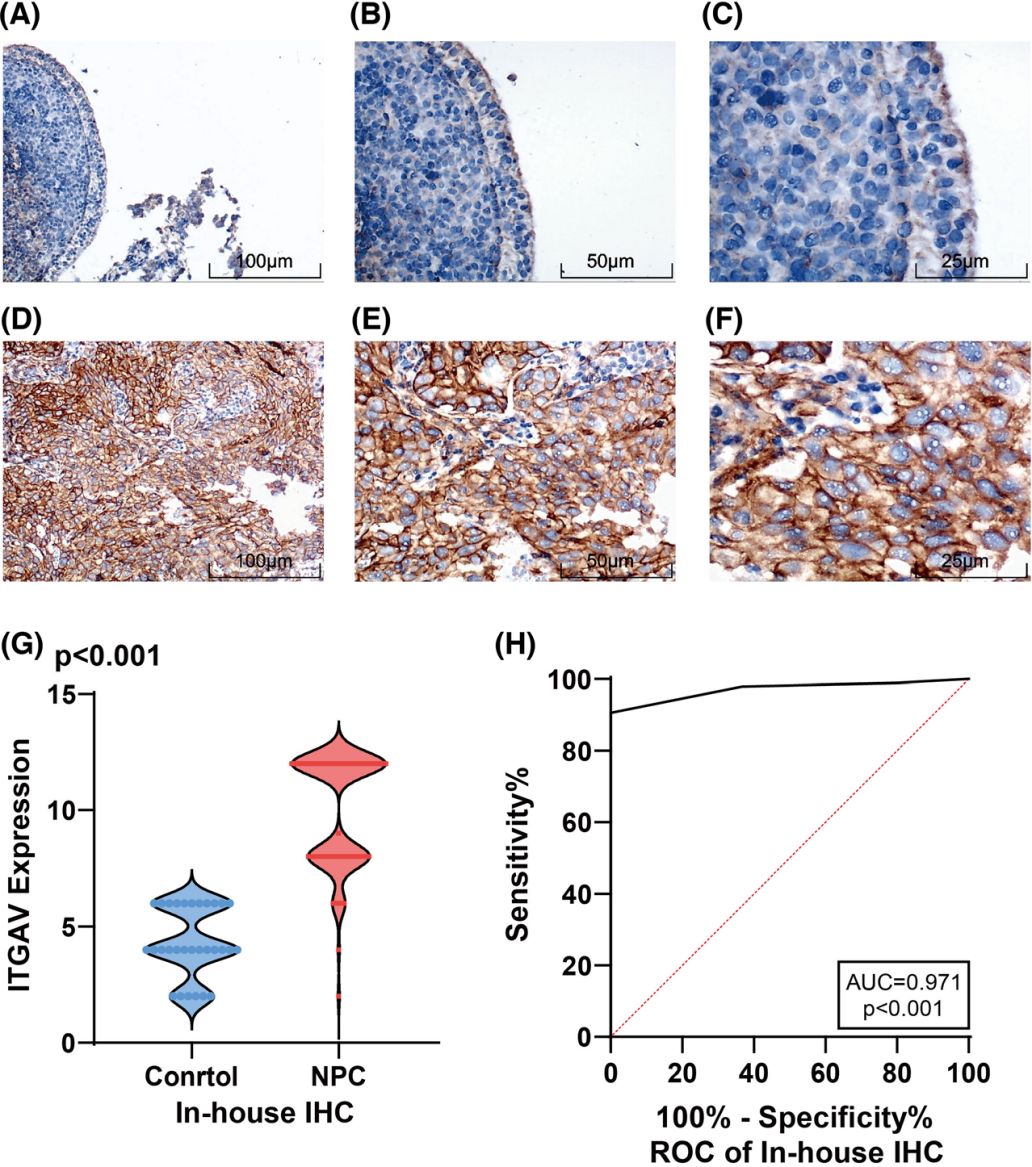
Expression of ITGAV protein in NPC. (A–C) ITGAV expression in non‐cancer tissues; (D–F) ITGAV expression in NPC tissues. Magnification: ×100 (A, D) (scale bar = 100 μm), ×200 (B, E) (scale bar = 50 μm) or ×400 (C, F) (scale bar = 25 μm); (G) the expression of ITGAV between non‐cancer and NPC based on IHC; (H) the ROC curve of ITGAV based on IHC. ROC, receiver operating characteristic curve.

**Fig. 8:**
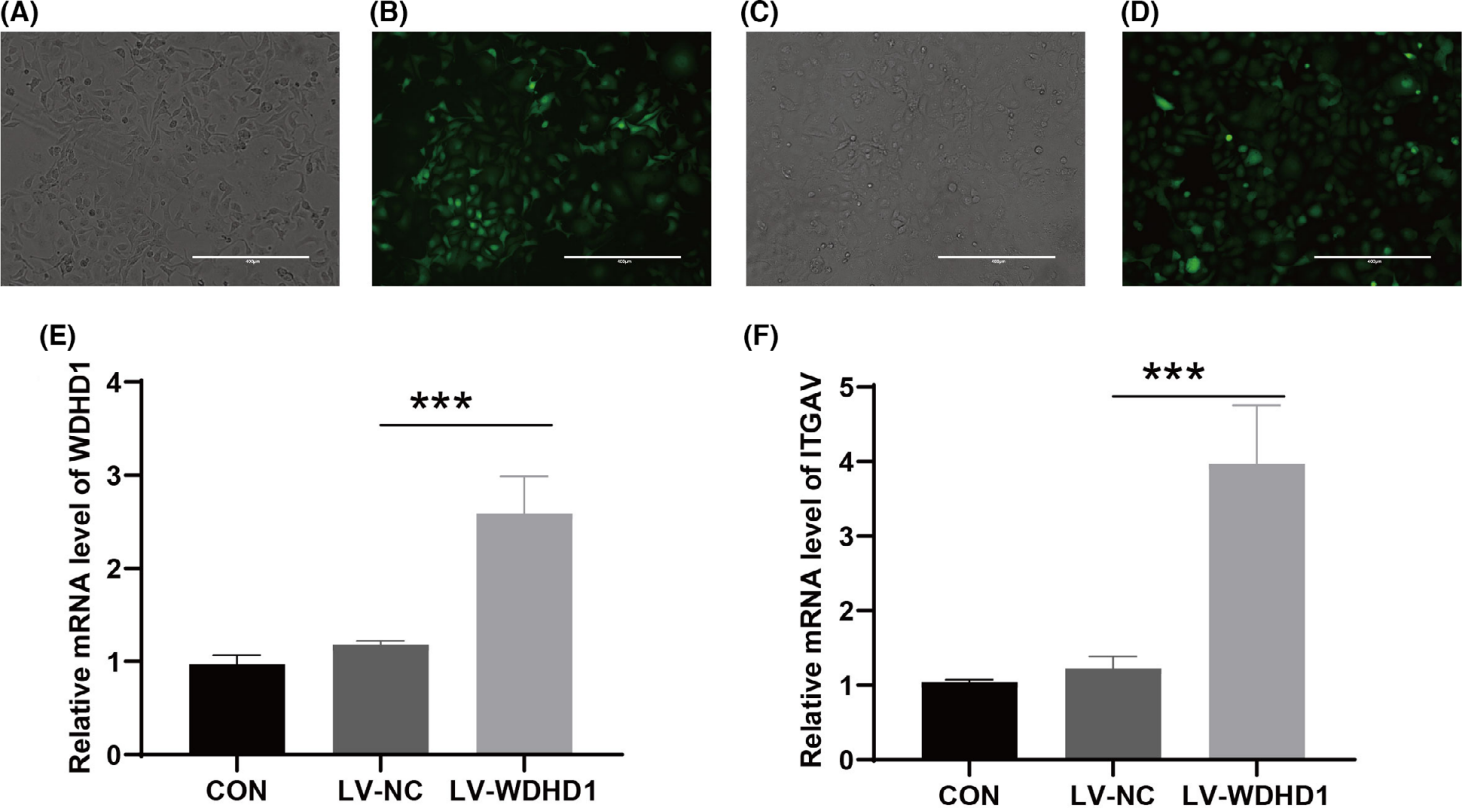
Detection of overexpress efficiency of WDHD1 gene. (A–D) Transfection fluorescence figures of C666‐1 cells observed under inverted fluorescence microscope (×100) (scale bar = 400 μm); (A, B) negative control group; (C, D) LV–WDHD1 group; (E) detection of overexpress efficiency of WDHD1 gene by RT‐qPCR; error bars show means ± standard deviation (SD; *n* = 3); (F) RT–qPCR detected the expression of ITGAV between the LV‐NC and the LV‐WDHD1; error bars show means ± SD (*n* = 3). ***, *P* < 0.001; *P*, *P* value of Student's *t*‐test.

We also explored the possible role of WDHD1 in NPC by screening NPC DEGs, WDHD1 positively related genes and target genes. According to the screening criteria, a total of 1115 up‐regulated DEGs, 6137 WDHD1 positively related genes, and 4112 target genes were screened. The intersection of the three gene sets was 181 (Fig. 4A; Table S1). For ITGAV‐related DEGs, there were 523 up‐regulated DEGs, and 1712 ITGAV positively related genes (Fig. 4B). We found that integrin alpha V (ITGAV) and WDHD1 had significant binding peaks upstream of the transcription start site (Fig. 5A) and that their expressions were significantly positively correlated (Fig. 5B–L). Extraction of the ITGAV expression data to clarify its mRNA expression level in NPCs revealed a higher expression level in NPC than in non‐NPC tissues (SMD = 2.01, 95% CI = 1.33–2.69); *I* ^2^ = 65.4% and *P* = 0.002, indicating significant heterogeneity, and a random effect model was chosen for analysis (Fig. 6A). The Egger test did not detect significant publication bias (*P* = 0.484, > 0.05) (Fig. 6B), and the sROC curve showed AUC = 0.99 [0.98–1.00], indicating a strong ability of ITGAV to distinguish NPC from non‐NPC tissue (Fig. 6C). Subsequent IHC confirmed that ITGAV was also highly expressed at the protein level (Fig. 7A–F), and the violin diagram shows this result more intuitively (Fig. 7G). The ROC curve suggested that ITGAV had a strong ability to distinguish between NPC and non‐NPC tissue (Fig. 7H). The results of PCR indicated that we had successfully constructed the WDHD1 overexpressed stable strains in C666‐1, the cell transfection efficiency rate of nearly 100% (Fig. 8A–D) and the mRNA expression level of WDHD1 was higher in the overexpressed group than in the control group (*P* < 0.05) (Fig. 8E). More importantly, we found that the expression of ITGAV increased after the overexpression of WDHD1 in C666‐1 (Fig. 8F). These results revealed that WDHD1 may affect the progression of NPC through the transcriptional regulation of ITGAV expression.

**Fig. 9:**
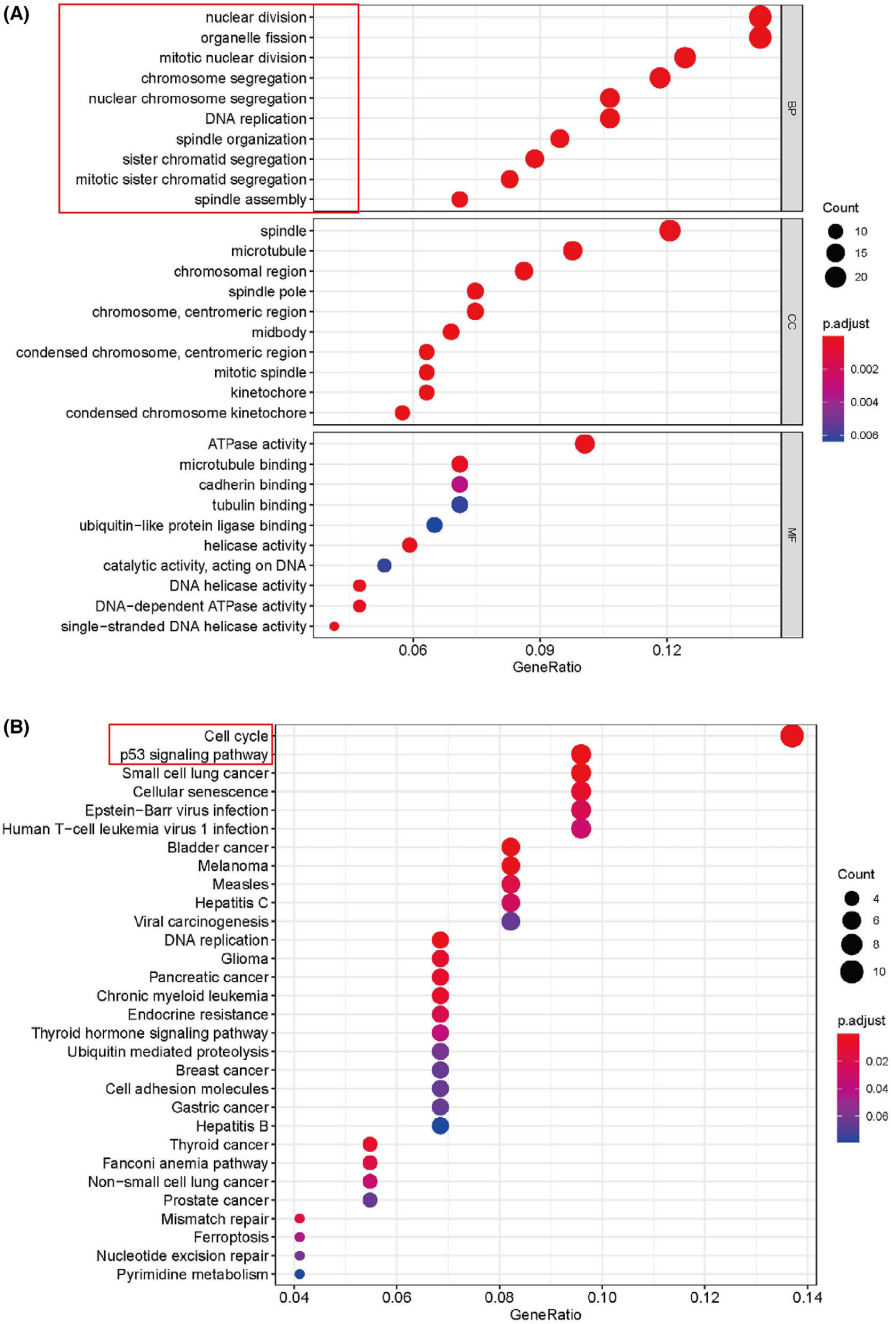
Part of the bubble chart for enriched GO and KEGG items of WDHD1 (*P* < 0.05). (A) GO enrichment; (B) KEGG pathways. *P* < 0.05 indicates significant difference.

**Fig. 10:**
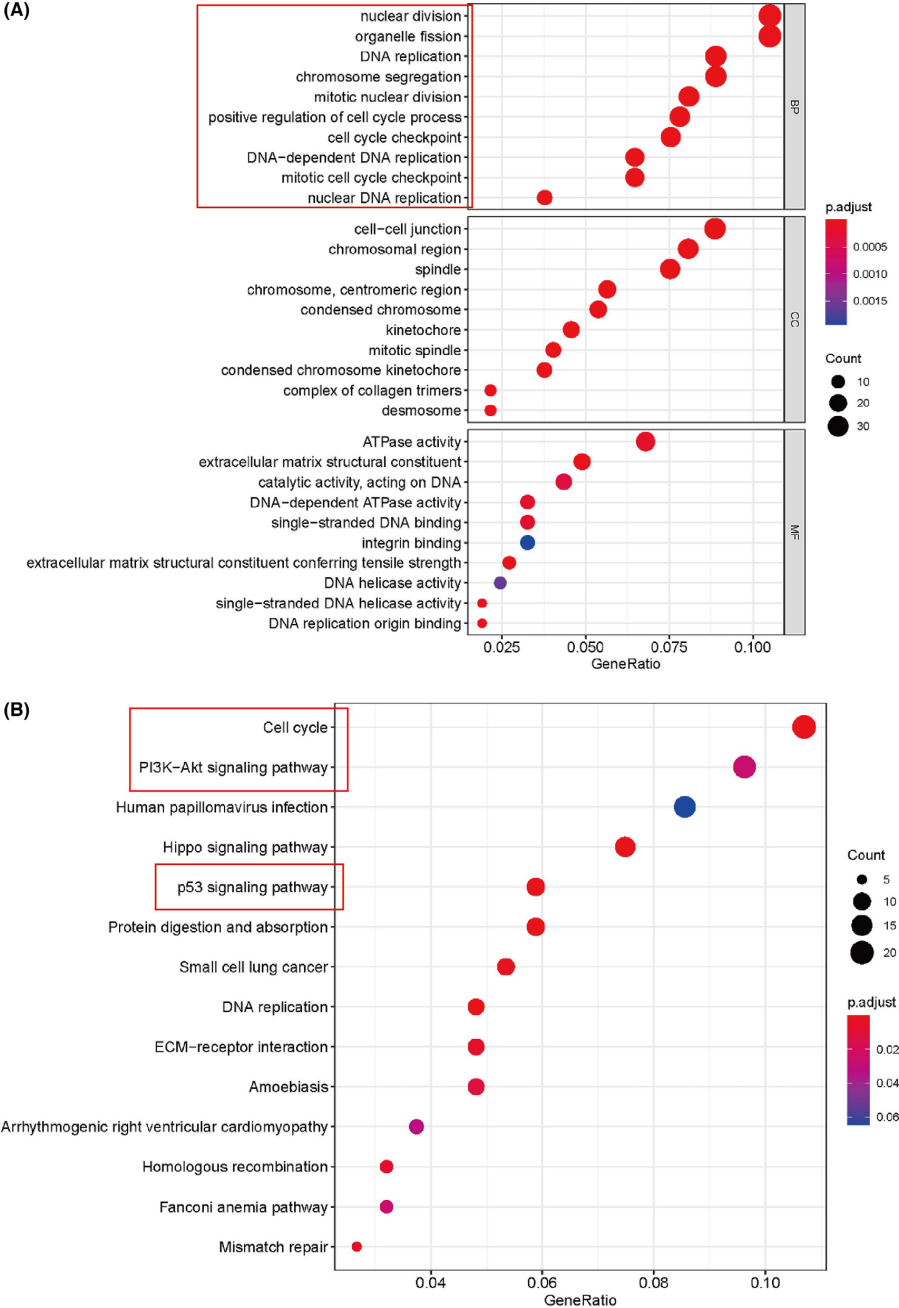
Part of the bubble chart for enriched GO and KEGG items of ITGAV (*P* < 0.05). (A) GO enrichment; (B) KEGG pathways. *P* < 0.05 indicates significant difference.

We further explored the cellular functions and biological processes of these two genes by conducting enrichment analyses. The first 10 records of the results are shown in Fig. 9. The GO analysis indicated that WDHD1‐related target genes were mainly enriched in functions related to the cell cycle, such as organelle fission, nuclear division, and DNA replication (*P* < 0.05). The KEGG enrichment analysis showed that these genes were closely connected with the cell cycle (*P* < 0.05). The GO functional items of the ITGAV‐related DEGs were enriched in organelle fission, nuclear division, DNA replication, and positive regulation of the cell cycle process (*P* < 0.05) (Fig. 10A), while the KEGG analysis revealed that these genes mainly participated in the cell cycle and PI3K–Akt signaling pathway (Fig. 10B). These enrichment results suggest that WDHD1 and ITGAV are closely related to the regulation of the cell cycle in NPC.

### WDHD1 is closely related to cell apoptosis

**Fig. 11:**
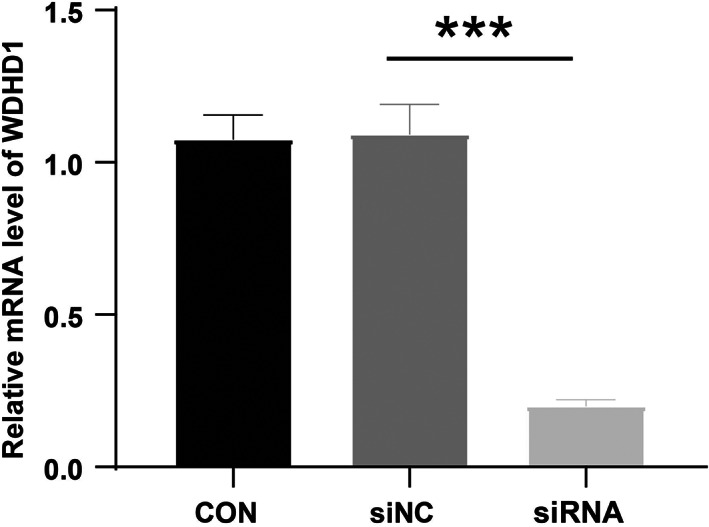
Detection of knockdown efficiency of WDHD1 gene by RT–qPCR. Error bars show means ± standard deviation (SD; *n* = 3); ***, *P* < 0.001; *P*, *P* value of Student's *t*‐test.

**Fig. 12:**
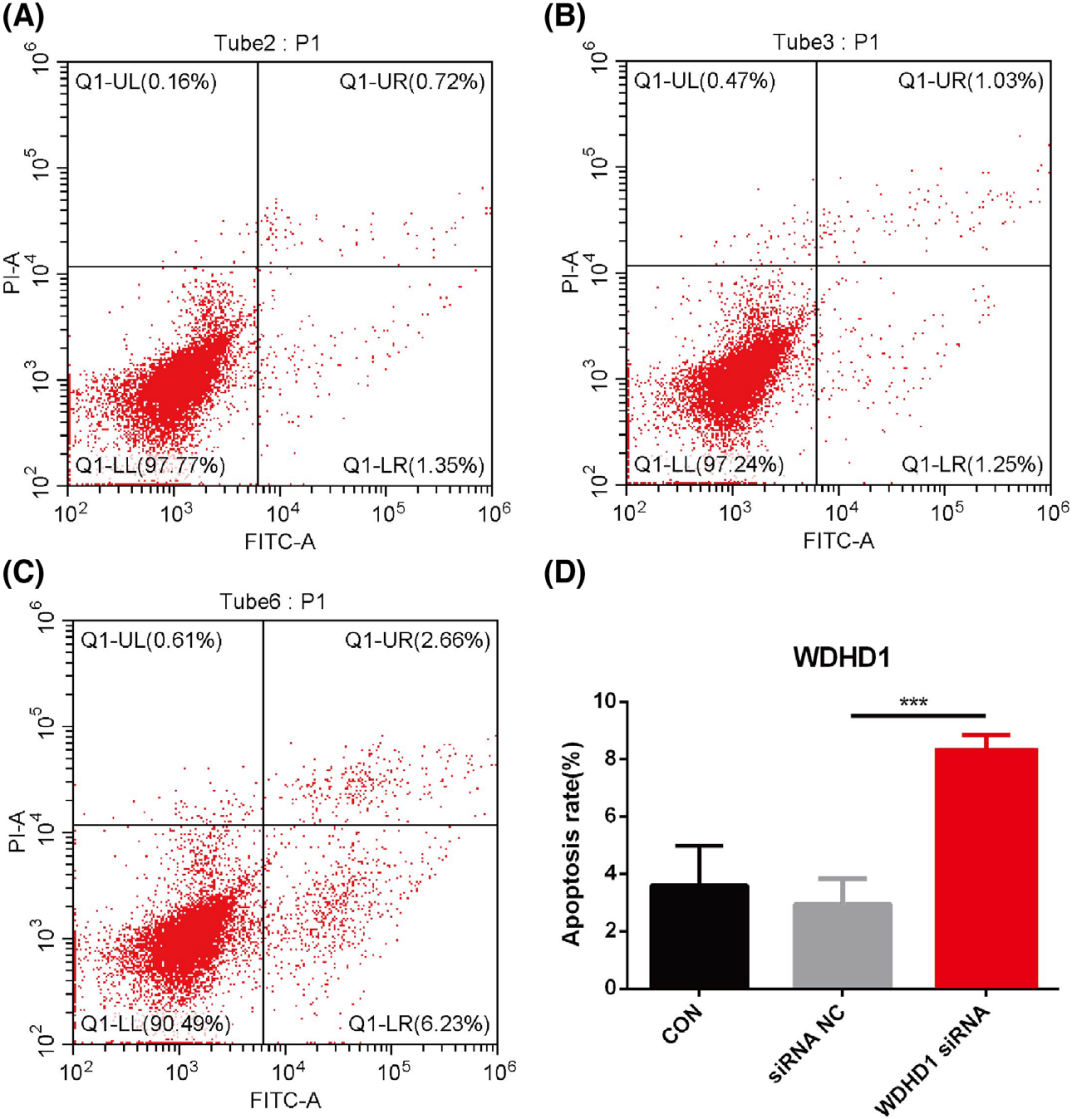
WDHD1 gene knockdown promotes apoptosis. (A) Control group; (B) negative control group; (C) WDHD1 siRNA group; (D) statistical analysis of apoptosis in each group in three independent experiments; error bars show means ± standard deviation (SD; *n* = 3). ***, *P* < 0.001; *P*, *P*‐value of Student's *t*‐test.

At 48 h after siRNA transfection, RT‐QPCR was used to detect the relative expression level of WDHD1 mRNA in CNE‐2 cells, and it was found that the expression level of WDHD1 mRNA in the interference group was lower than that in the control group (*P* < 0.05) (Fig. 11). Subsequently, we conducted a more in‐depth study of the mechanism of WDHD1 in NPC by flow cytometry of CNE‐2 cells that were successfully transfected with WDHD1 siRNA. The numbers of early and late apoptotic cells increased significantly after knockdown of WDHD1 expression with siRNA (NC: 2.93 ± 0.88%; WDHD1 siRNA: 8.31 ± 0.54%) (Fig. 12).

## Discussion

The WDHD1 is a DNA‐binding protein containing the WD40 domain and an “HMG” box, which is closely related to the post‐transcriptional steps of the centromeric silencing pathway, homologous recombination repair, and the assembly and activation of the pre‐replication complex (pre‐RC), two key steps in the initiation of DNA replication [7, 14, 15]. It plays a significant role in regulating protein signal transduction, transcription, cell proliferation, and apoptosis [16]. A number of existing studies have shown that WDHD1 is closely related to tumor progression. For example, Liu et al. [11] found that miR‐494 may play a role in epithelial‐mesenchymal transformation (EMT), tumor growth and metastasis of cholangiocarcinoma by negatively regulating WDHD1, and Zhou and Chen [17] demonstrated that STAT3 may affect DNA replication through transcriptional regulation of WDHD1. He et al. [18] analyzed the expression of WDHD1 in hepatocellular carcinoma (HCC) by combining IHC and bioassay, and found that WDHD1 was highly expressed in HCC and may be associated with its poor prognosis. Our research group was surprised to find that WDHD1 was also highly expressed in laryngeal squamous cell carcinoma [19]. In a recent study, Gou et al. knocked down the expression of WDHD1, tested the radiotherapy sensitivity of non‐small cell lung cancer cells (NSCLC) by clonal formation assay, flow cytometry, comet assay, and immunofluorescence and verified it in a vivo xenograft tumor model. The results showed that knocking down the expression of WDHD1 significantly increased the radiotherapy sensitivity of NSCLC [20]. Despite these studies, its role in NPC has not been reported. In our study, we first combined high‐throughput data with immunohistochemistry, followed by prediction of the biological function of WDHD1 and its target genes with GO and KEGG enrichment analysis, then preliminary verification using flow apoptosis assays, and finally an evaluation of WDHD1 in NPC to determine its possible molecular mechanism. To the best of our knowledge, this is the first study to verify the expression level of WDHD1 in NPC by combining multiple methods. It is also innovative in its use of pathway analysis, PCR and flow cytometry detection of apoptosis to explore the molecular mechanism of WDHD1 as a transcription factor in the malignant progression of NPC.

The bioprediction and immunohistochemistry results presented here indicate that WDHD1 is significantly overexpressed in NPC in most datasets. The forest map showed SMD = 1.22 (95% CI = 0.40–2.04) and *I* ^2^ = 79.3%, indicating significant heterogeneity. This may reflect the fact that the data samples come from different countries or that the microarray platforms differ. The reason for the significantly higher expression of WDHD1 in the control group than in the experimental group in GPL11154 (platform merged datasets) might be that some of the included datasets had no control group or that the sample size difference between the two groups was too large. Enrichment analysis suggested that WDHD1 was closely related to cell cycle functions, which was further verified by the results of the flow cytometry apoptosis assay.

We also evaluated the mechanism by combining the WDHD1 target genes for analysis. The promoter of the ITGAV gene is a DNA sequence that RNA polymerase identifies, binds to, and uses to initiate transcription. ITGAV showed an obvious peak with WDHD1 and was significantly positively correlated with WDHD1 expression. PCR indicated that the expression of ITGAV increased with the increase of WDHD1 in C666‐1. Therefore, we speculated that WDHD1 regulates the cell cycle by transcriptional regulation of ITGAV expression. Integrin is a kind of transmembrane glycoprotein receptor that binds cells and the matrix. It is composed of an α subunit, a β subunit and bidirectional signaling molecules, and participates in cell adhesion and differentiation. ITGAV is located on chromosome 2q31‐q32, and as a protein‐coding gene, it is closely related to tumor angiogenesis, invasion and metastasis. Existing studies have shown that ITGAV overexpression is related to the progression of some tumors, such as glioblastoma and laryngeal, breast, esophageal, and colorectal cancers [21, 22, 23].

With regard to the relationship between ITGAV and NPC, Ding et al. [24] demonstrated experimentally that ITGAV is the target gene of miR‐9‐3p, which can promote the proliferation and metastasis of NPC cells by promoting the EMT process. In our research, ITGAV was significantly overexpressed in NPC tissues and was closely related to the p53 and PI3K/Akt signaling pathways. The p53 gene is the most commonly mutated gene in human cancers and was initially considered to be an oncogene; however, subsequent studies have revealed that the wild‐type p53 gene can inhibit tumor growth and cell canceration [25, 26]. At present, many studies have found that the p53 signaling pathway plays an important role in tumor proliferation [27, 28]. The PI3K/Akt signaling pathway is recognized as closely related to cell apoptosis, tumor angiogenesis, and the cell cycle. Many studies have shown that this pathway is abnormally activated in a variety of malignant tumours, such as liver, gastrointestinal, and pancreatic cancers, and regulates cell proliferation, survival, transformation and other functions [29, 30, 31]. Therefore, we speculate that WDHD1 plays a role in the malignant progression of NPC through transcriptional regulation of ITGAV expression and subsequent regulation of the p53 or PI3K/Akt signaling pathways. However, this possibility requires further experimental verification, and our research group is currently conducting relevant studies.

In the present study, a role for WDHD1 in the malignant progression of NPC was evident, although the study had some limitations. One was that the heterogeneity among the included studies was large, which we speculate was due to a number of reasons: (a) some of the data samples came from different countries or the microarray platforms differed; (b) in some studies, there was a large difference in the number of samples between the control group and the experimental group, and some had no control group at all; and (c) the sample sources were different. For the experimental group, the sample sources included NPC biopsy, primary tumor, and cell lines. For the control tissues, the sources included normal nasopharyngeal mucosa, inflamed nasopharyngeal mucosa, and primary tonsillar epithelial cells. Another limitation is that the number of included specimens was small, so further expansion of the sample size is needed to reduce sample size errors. A third limitation is that the results presented here are derived mainly from bioprediction, so they lack full *in vivo* and *in vitro* verification. Our future goal is to conduct *in vitro* and *in vivo* experiments to further improve this study.

Overall, we found that WDHD1 is highly expressed in NPC and may influence the NPC cell cycle by regulating ITGAV expression. Bioinformatic analysis showed that WDHD1 has potential value in differentiating an NPC group from a non‐cancer group and may be useful as a new NPC screening molecule. Therefore, this research may serve as the basis for developing new diagnostic and therapeutic targets for the treatment of NPC.

## Conflict of interest

The authors declare no conflict of interest.

## Author contributions

J‐YW and Y‐TN were in charge of drafting the work and performing most experiments; S‐NH, Y‐MT, Z‐DY and Y‐YF performed Immunohistochemical test; LJ, T‐TZ and X‐FZ performed qPCR and flow cytometry; J‐YW, Y‐XP, MM and C‐XL were accountable for analyzing the data and drafting figures; Z‐XW supervised the project and modified the manuscript.

## Supporting information


**Table S1.** A total of 181 WDHD1 positively related differentially expressed target genes are obtained.

## Data Availability

All data during this study are included within this published article and additional files. Any material described in the article can be requested directly from corresponding author on reasonable request. All the data in the current study are based on public data available in the Gene Expression Omnibus (GEO) datasets.
